# Gene Expression Changes in the Olfactory Bulb of Mice Induced by Exposure to Diesel Exhaust Are Dependent on Animal Rearing Environment

**DOI:** 10.1371/journal.pone.0070145

**Published:** 2013-08-05

**Authors:** Satoshi Yokota, Hiroshi Hori, Masakazu Umezawa, Natsuko Kubota, Rikio Niki, Shinya Yanagita, Ken Takeda

**Affiliations:** 1 Department of Hygiene Chemistry, Faculty of Pharmaceutical Sciences, Tokyo University of Science, 2641 Yamazaki, Noda, Chiba, Japan; 2 The Center for Environmental Health Science for the Next Generation, Research Institute for Science and Technology, Tokyo University of Science, 2641 Yamazaki, Noda, Chiba, Japan; 3 Faculty of Science and Technology, Tokyo University of Science, 2641 Yamazaki, Noda, Chiba, Japan; School of Biomedical Sciences, The University of Queensland, Australia

## Abstract

There is an emerging concern that particulate air pollution increases the risk of cranial nerve disease onset. Small nanoparticles, mainly derived from diesel exhaust particles reach the olfactory bulb by their nasal depositions. It has been reported that diesel exhaust inhalation causes inflammation of the olfactory bulb and other brain regions. However, these toxicological studies have not evaluated animal rearing environment. We hypothesized that rearing environment can change mice phenotypes and thus might alter toxicological study results. In this study, we exposed mice to diesel exhaust inhalation at 90 µg/m^3^, 8 hours/day, for 28 consecutive days after rearing in a standard cage or environmental enrichment conditions. Microarray analysis found that expression levels of 112 genes were changed by diesel exhaust inhalation. Functional analysis using Gene Ontology revealed that the dysregulated genes were involved in inflammation and immune response. This result was supported by pathway analysis. Quantitative RT-PCR analysis confirmed 10 genes. Interestingly, background gene expression of the olfactory bulb of mice reared in a standard cage environment was changed by diesel exhaust inhalation, whereas there was no significant effect of diesel exhaust exposure on gene expression levels of mice reared with environmental enrichment. The results indicate for the first time that the effect of diesel exhaust exposure on gene expression of the olfactory bulb was influenced by rearing environment. Rearing environment, such as environmental enrichment, may be an important contributive factor to causation in evaluating still undefined toxic environmental substances such as diesel exhaust.

## Introduction

Diesel exhaust (DE) consists of a complex mixture of components in gaseous or particulate form (DEP: diesel exhaust particles). DEP comprise more than 1,000 chemicals that are mainly composed of cores of elemental carbon, traces of metallic compounds, and adsorbed organic materials including polycyclic aromatic hydrocarbons, aldehydes, and nitrogen oxides [1]. DEP accumulate and negatively affect lung function following inhalation exposure. It has been reported that DE causes lung cancer [2], allergic rhinitis [3], and bronchial asthma-like diseases [4]. In fact, the International Agency for Research on Cancer, which is part of the World Health Organization, classified DE as carcinogenic to humans (Group 1) in 2012, based on sufficient evidence that DE exposure is associated with an increased risk of lung cancer [5].

Some research has led to the concern that the brain represents a target for the effects of ambient particulate matter (PM), mainly derived from DEP in urban environments [6]. It has been demonstrated that a small fraction of inhaled nanoparticles may reach the brain [7], [8]. Nanoparticles deposit in alveolar and subsequently cross the lung–blood barrier and the blood–brain barrier, and small nanoparticles, especially those below 10 nm, deposit efficiently on the olfactory mucosa by diffusion. Subsequent uptake and translocation of nanoparticles along axons of olfactory nerves has been reported [9], [10]. Previous *in vitro* studies have demonstrated that nanoparticles, such as DEP, might cause neurotoxic effects and disturb blood–brain barrier function [11], [12]. In addition, *in vivo* studies also have demonstrated that DE inhalation exposure has inflammatory effects on the olfactory bulb and midbrain regions of the brain [13], [14].

However, these toxicological studies have not evaluated the effects of conditions in the animal rearing environment. Effects of exposure to environmental chemicals and materials including nanoparticles are usually assessed in a standard laboratory cage environment alone. Mice housed in standard laboratory cages demonstrate frequent stereotyped behavior, in particular spontaneous jumping and backward flipping, which is persistent and occurs early in development compared with those housed in environmental enrichment conditions [15]. In contrast, environmental enrichment, which provides a combination of complex inanimate and social stimulation, may improve the well-being of animals reared in standard housing [16]. It has been reported that environmental enrichment attenuated stereotyped behavior compared with a standard laboratory housing environment [17]. Stereotyped behavior is a feature of autism [18] and is often observed in persons with mental retardation or developmental disorders [19], [20], [21]. Standard housing environment may change mice phenotypes [22] and thus might alter toxicological research results.

We hypothesized that chemical substance exposure-induced toxicity might be influenced by housing environment. In fact, it has been recommended that rodents reared in environmental enrichment should be used for regulatory toxicological research [23]. In particular, the rearing environment during the perinatal period is important for the development of the central nervous system of offspring [24], which suggests the possibility that early rearing environment might change the susceptibility of animals to chemical substance exposure. However, there are no data that have investigated if early rearing environment alters the later effects of DE exposure on the central nervous system.

We focused on the olfactory bulb because the olfactory translocation route is one of the targets of DEP [25]. The objective of the present study was to gain insight into how gene expression changes in the olfactory bulb of mice reared in a standard cage environment compare with those reared in environmental enrichment during the perinatal period when they were exposed to DE.

## Materials and Methods

### Animals

Pregnant C57BL/6J mice, weighing approximately 30 g (Figure S1A) at gestational day 14, were purchased from CLEA Japan, Inc. (Tokyo, Japan) and used for experiments. All animals were acclimated to our animal room (The Center for Environmental Health Science for the Next Generation, Research Institute for Science and Technology, Tokyo University of Science). They had free access to water and standard animal food and were exposed to a 12-hour light/dark cycle (lights on between 8∶00 and 20∶00), a temperature of 22±1°C, and a humidity-controlled environment (50±5%). Body weights of dams and their pups were recorded at sampling (Figure S1B–D). All experiments were performed in accordance with Animal Research: Reporting In Vivo Experiments guidelines for the care and use of laboratory animals [26] and were approved by Tokyo University of Science’s Institutional Animal Care and Use Committee. All sampling was performed under sodium pentobarbital (50 mg/kg) anesthesia, and all efforts were made to minimize suffering.

### Housing Environmental Conditions

**Figure 1:**
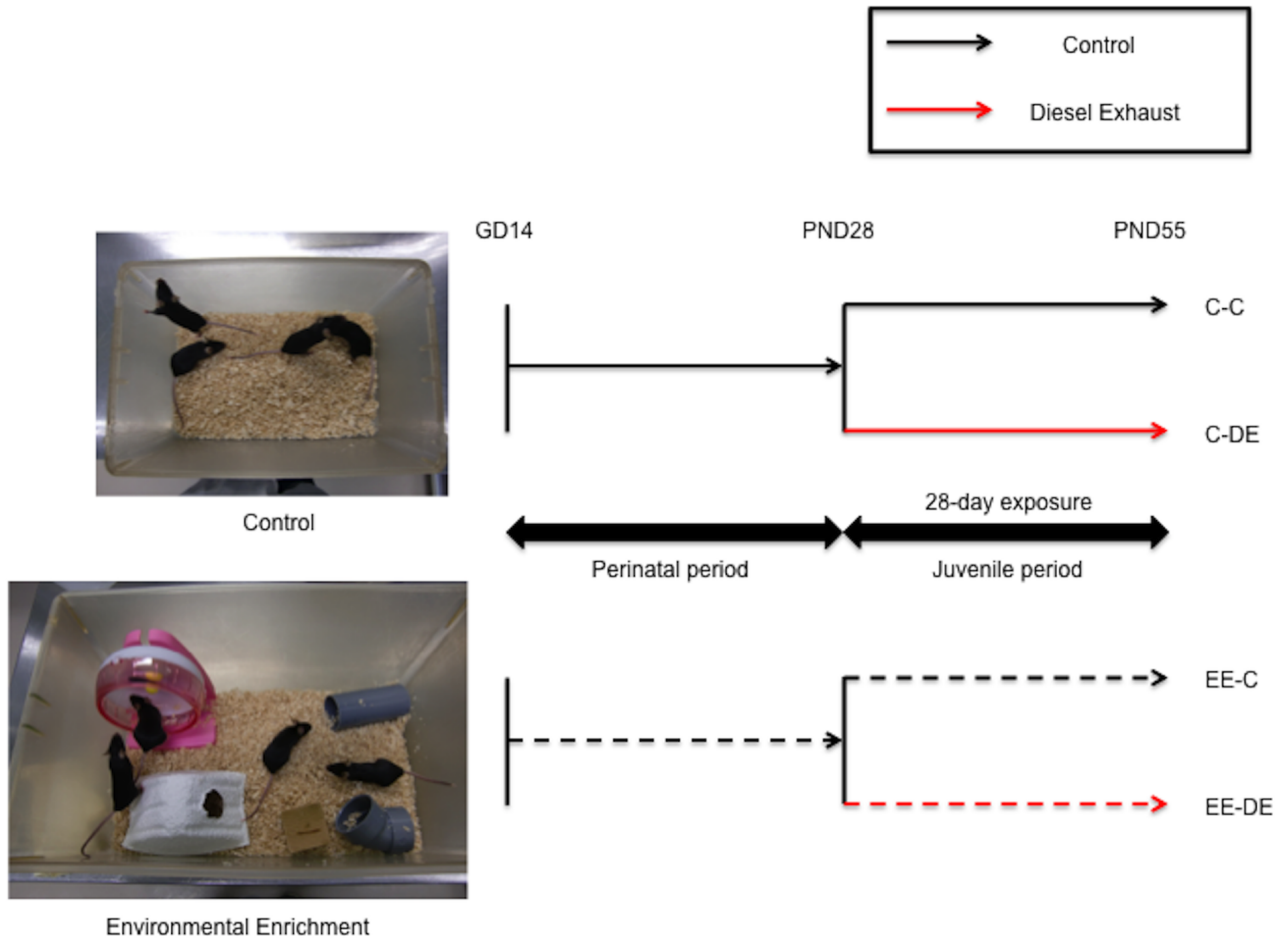
Schematic diagram of design of the present study.

Upon arrival to the colony, half of the 20 pregnant dams were assigned to the standard cage environment (C) and the other half to environmental enrichment (EE). Standard laboratory cage consisted of a common housing cage for mice (30×20×12.5 cm: 7500 cm^3^). Environmental enrichment consisted of a larger (40×25×19 cm: 19,000 cm^3^) cage containing a running wheel, small house, wood blocks, and plastic tubing that were moved to different locations within each cage every 2–3 days and were exchanged with new toys. One dam and her pups (n = 8) were housed in either the standard laboratory cage or environmental enrichment throughout the perinatal period and until weaning. After weaning at postnatal day 27, the offspring mice were placed in a control chamber or DE inhalation chamber and were housed under the same conditions as during the perinatal period. The mice were exposed to DE for 8 hours/day (10∶00–18∶00) for 28 days (postnatal days 28−55) in the control or DE inhalation chamber at the Center for Environmental Health Science for the Next Generation (Research Institute for Science and Technology, Tokyo University of Science). Housing environment (standard cage environment [C] or environmental enrichment [EE]) during the perinatal period (gestational day 14–postnatal day 28) and chamber (control [C] and [DE]) established four experimental groups: C-C, C-DE, EE-C, and EE-DE (Figure 1). Necropsies were performed 1 day after the final exposure. In this experiment, 7–9 independent litters (C-C: n = 7, C-DE: n = 7, EE-C: n = 8, EE-DE: n = 9) were used. The olfactory bulb was collected from each male mouse, frozen quickly in liquid nitrogen, and then stored at −80°C until total RNA extraction. Lung tissues were also collected and immersed in 10% phosphate buffered formalin until use.

### Diesel Exhaust

A four-cylinder 2,179 cc diesel engine (Isuzu Motors Ltd., Tokyo, Japan) was operated at a speed of 1500 rpm and 80% load with diesel fuel. The exhaust was introduced into a stainless steel dilution tunnel (216.3 mm diameter × 5250 mm) where the exhaust was mixed with clean air. Particle size distributions (measuring range 10–410 nm) were investigated using a scanning mobility particle sizer apparatus (model 3936, TSI Inc., St. Paul, MN, USA) composed of a condensation particle counter (model 3785, TSI Inc.) and a differential mobility analyzer (model 3081, TSI Inc.). The apparatus was operated at a sample flow rate of 0.6 L/minute and a sheath flow rate of 6.0 L/minute. The mass and number concentrations of DEP were measured by a Piezobalance Dust Monitor (model 3521, Kanomax, Inc., Osaka, Japan) and a condensation particle counter (model 3007, TSI Inc.), respectively. Concentrations of gas components, [i.e., nitric oxide (NO_x_), sulfur dioxide (SO_2_), and carbon monoxide (CO)] in the chambers were measured by an NO-NO_2_-NO_x_ analyzer model 42i (Thermo Fisher Scientific Inc., Franklin, MA, USA), Enhanced Trace Level SO_2_ Analyzer, Model 43i-TLE (Thermo Fisher Scientific Inc.), and a CO Analyzer, model 48i (Thermo Fisher Scientific Inc.), respectively.

### Immunohistochemistry

Dams at the weaning period were perfused transcardially with a heparin solution (1000 U/l, 0.9% saline), followed by ice-cooled fixative composed of 4% paraformaldehyde, 0.1% glutaraldehyde, and 0.2% picric acid in 0.1 M phosphate buffered saline (PBS, pH 7.4). The brains were removed and post-fixed in the same fixative without glutaraldehyde for 24 hours at 4°C. The brains were then cryoprotected in a phosphate-buffered 30% sucrose solution with 0.1% sodium azide for 24–48 hours. The brains were then frozen and cut in the coronal plane (6 series of 40- µm thick sections) on a microtome (Sakura Finetek Co., Ltd., Japan) and collected in 0.1 M PBS with 0.1% sodium azide.

Immunohistochemical visualization of FosB was performed on free-floating sections using antibody and avidin-biotin peroxidase methods as previously described [27], [28]. Briefly, after blocking endogenous peroxidase and preincubation in 10% normal horse serum, the sections were incubated in primary rabbit polyclonal affinity purified anti-FosB antibody (sc-48, Santa Cruz Biotechnology, Inc., Santa Cruz, CA, USA) diluted 1∶600 in 0.1 M PBS with 0.1% Triton X-100 for 16 hours at room temperature. After three 10-minute rinses in 0.1 M PBS with 0.1% Triton X-100, the sections were further incubated in a biotinylated secondary antibody solution, donkey anti-rabbit IgG (AP182B, Chemicon, Temecula, CA, USA, 1∶800) for 120 minutes at room temperature, followed by three 10-minute rinses in 0.1 M PBS with 0.1% Triton X-100, and finally treated with an avidin-biotin peroxidase complex (Vectastain ABC peroxidase kit, Vector Laboratories Inc., Burlingame, CA, USA, 1∶400) for 240 minutes. The sections were reacted for peroxidase activity in a solution consisting of nickel ammonium sulfate, 0.02% 3,3-diaminobenzidine in 0.1 M Tris-HCl buffer (pH 7.6), and 0.01% H_2_O_2_ for 20 minutes. FosB immunoreactivity was localized to the cell nuclei and appeared as a dark gray-black stain. Subsequently, sections were washed in 0.01 M PBS, mounted on gelatin-coated glass slides, air-dried, dehydrated in a graded series of alcohols, cleared in xylene, and coverslipped with Entellan (Merck Co, Ltd., Japan). Photomicrographs were captured with a light microscope (BX51; Olympus Co., Ltd., Japan).

### Hematoxylin-eosin Staining

Lung tissues were embedded in paraffin, cut on the microtome, and then stretched in a water bath. Paraffin-embedded lung sections were removed from the water bath and air-dried for 1 hour at 40°C on slides. Staining was performed manually in staining dishes as follows: a de-washing step in xylene, then rehydration with successive incubations in 95% ethanol, and finally tap water. Hematoxylin 3G (Sakura Finetek Co., Ltd., Japan) was applied for 5 minutes followed by a wash with running tap water for 5 minutes and staining with eosin (Sakura Finetek Co., Ltd., Japan) for 3 minutes. After washing with tap water and dehydration with successive washes of 95% ethanol and xylene, slides were mounted by Entellan and air-dried prior to microscopic examination.

### Total RNA Isolation

Olfactory bulbs were immediately isolated (within 50 seconds), frozen in liquid nitrogen, and kept at −80°C. Total RNA was isolated using Isogen (Nippon Gene Co., Ltd., Tokyo, Japan) according to the manufacturer’s protocol and suspended in pure water. The RNA quantity was determined by spectrophotometry measurement of OD260/280 (ratio >1.8) in a BioPhotometer plus (Eppendorf, Hamburg, Germany). Extracted RNA from each sample was used for microarray and quantitative RT-PCR analysis.

### Complementary DNA Microarray Procedures

After purification of RNA by ethanol precipitation and an RNeasy Micro Kit (Qiagen, Hilden, Germany), the RNA integrity was evaluated by capillary electrophoresis using a Bioanalyzer 2100 (Agilent Technologies, Inc., Santa Clara, CA, USA). Each RNA sample (31 individual samples) showed 8.8−9.9 in the RNA integrity number scores. To reduce false positives due to variability between individual samples, equal amounts of total RNAs from individual samples from each cage (one olfactory bulb sample/cage) were pooled (7−9 sample pooling in each group). To improve the accuracy of the data, the pooled RNA template was divided into two replicates for technical analysis. In this pooled data set, the average data between two replicate arrays were used for microarray analysis. Each of the pooled RNA samples was labeled with Cy3 and hybridized to a SurePrint G3 Mouse GE 8×60K microarray (Agilent Technologies) consisting of 62,976 spots (28,620 genes) according to the protocol of DNA Chip Research Inc. (Kanagawa, Japan). After hybridization with fluorescent-labeled cDNA, the microarray was washed using Gene Expression Wash Buffers Pack (Agilent Technologies) and then scanned by a DNA microarray Scanner G2565CA (Agilent Technologies). Scanner output images were normalized and digitalized by Agilent Feature Extraction software according to the Minimum Information about a Microarray Experiment guidelines [29] and a pre-processing method for Agilent data [30]. The raw data and normalized data have been deposited in NCBI’s Gene Expression Omnibus and are accessible thorough Gene Expression Omnibus Series accession number GSE46163 (http://www.ncbi.nlm.nih.gov/geo/query/acc.cgi?acc=GSE46163).

### Hierarchical Cluster Analysis

To extract characteristic gene sets that were differentially expressed after subacute exposure to DE, the log_2_ fold-change data (C-C vs. C-DE, EE-C vs. EE-DE, and C-C vs. EE-DE comparison in offspring) of gene expression were hierarchically clustered using a complete linkage algorithm and Euclidean distance as the distance metric [31]. The analysis was performed using Cluster 3.0 [32], and the result was visualized by Java TreeView [33].

### Defining Functional Relationships between Expression Profiles

To better understand the biological meanings of the microarray results, functional analyses were performed using gene annotation by Gene Ontology (GO) and canonical pathway analysis. All genes printed on the microarray were annotated with GO using an annotation file (ftp://ftp.ncbi.nih.gov/gene/DATA/gene2go.gz) provided by National Center for Biotechnology Information (NCBI; Bethesda, MD) and pathway analysis using c2.cp.v3.1.symbols.gmt in http://www.broadinstitute.org/gsea/downloads.jsp#msigdb provided by Broad Institute (BI; Cambridge, MA). The annotation was updated in January 2013. All of the differentially expressed genes were classified by GO and pathway according to their function. In addition, some gene sets obtained by hierarchical cluster analysis were also categorized by GO. Enrichment factors for each GO and pathway were defined as (nf/n)/(Nf/N), where nf is the number of flagged genes within the category, Nf is the total number of genes within that same category, n is the number of flagged genes on the entire microarray, and N is the total number of genes on the microarray. Statistical analysis was performed using Fisher’s exact test based on a hypergeometric distribution to calculate P values. The categories with a high enrichment factor and P<0.05 were extracted.

### Quantitative RT-PCR

Total RNA (1 µg) for each sample was used as a template to synthesize cDNA using M-MLV reverse transcriptase (Invitrogen Co., Carlsbad, CA, USA) according to the manufacturer’s instructions. Quantitative Real-Time PCR (RT-PCR) was performed with SYBR Green Real-Time PCR Master Mix (Toyobo Co., Ltd., Osaka Japan., Thunderbird) in an Mx3000P (Agilent Technologies) with an initial hold step (95°C for 60 seconds) and 40 cycles of a two-step PCR (95°C for 15 seconds and 60°C for 60 seconds). At each cycle, the fluorescence intensity of each sample was measured to monitor amplification of the target gene. Relative expression levels of target genes were calculated for each sample after normalization against glyceraldehyde-3-phosphate dehydrogenase (*Gapdh*). We found no significant differences in the *Gapdh* expression between groups (data not shown). The primer sequences are shown in Table 1.

**Table 1:** Design of primer pairs for Real-Time RT-PCR analysis.

Gene symbol		Sequence (5′>3′)	Tm (°C)	GenBank Accession
*Gapdh*	Forward:	TGTGCAGTGCCAGCCTCGTC	60	NM_008084
	Reverse:	GGATGCATTGCTGACAATCT		
*Dbp*	Forward:	AAGCATTCCAGGCCATGAGAC	60	NM_016974
	Reverse:	CGGCTCCAGTACTTCTCATC		
*Cxcl10*	Forward:	CCGGAAGCCTCCCCATCAGC	60	NM_021274
	Reverse:	GGGATCCCTTGAGTCCCACTCAGAC		
*Chmp4b*	Forward:	GATGGCACCCTGTCAACCATC	60	NM_029362
	Reverse:	TGAGCTCATCCTCGTCGAAC		
*Fam13c*	Forward:	AGCTGAAGCTGTCGGAAGAGC	60	NM_024244
	Reverse:	GATACCTCTGCATCGGTCATA		
*Mslnl*	Forward:	GGCTTACTGTCATGCAGACTG	60	NM_177822
	Reverse:	AAGTGGCCTTGGACTCTAGG		
*Umodl1*	Forward:	AACTATAGCGTGTCCGCCAG	60	NM_177465
	Reverse:	TGCAGTGCAGGTAGACGATG		
*Aqp3*	Forward:	ATCTATGCACTGGCACAGAC	60	NM_016689
	Reverse:	ATTGACCATGTCCAAGTGTC		
*Cyp2f2*	Forward:	GAAGTCGCTTCGACTATGAC	60	NM_007817
	Reverse:	TCCATATTGAAGTGGCTCAG		
*Krt18*	Forward:	AGATTGCCAGCTCTGGATTG	60	NM_010664
	Reverse:	TGGTGACAACTGTGGTACTC		
*Sdad1*	Forward:	TGCTGCAGAACTTCATGTAC	60	NM_172713
	Reverse:	CACGCAGTTGTGATAACATTG		
*Nr1i3*	Forward:	TGCTACAAGATGGAGGACGC	60	NM_009803
	Reverse:	TTGTTCAGAATCAGCGCCATC		

Table 1 shows the gene symbol, primer pair sequences, T_m_, and GenBank accession numbers for the corresponding genes.

Footnote: T_m_ is the melting temperature of the PCR product.

### Statistical Analysis

We used 31 independent litters from 16 different rearing cages (standard rearing environment [7 independent cages] or environmental enrichment [9 independent cages]) during the perinatal period. Independent litters were composed of one pup from each dam from the control or environmental enrichment groups. The statistics were performed with the independent litter as the statistical unit. Values for body weight, food intake, immunohistochemistry, and quantitative RT-PCR are presented as the mean ± standard deviation (S.D.) One-way analysis of variance (ANOVA) followed by a subsequent simple-effects analysis with Tukey-Kramer multiple comparison test were used to determine differences between the different groups for food intake. Two-way ANOVA was used to evaluate DE exposure and rearing environment interaction effects for dependent variables. A significant interaction was interpreted by a subsequent simple-effects analysis with Tukey-Kramer multiple comparison test for quantitative RT-PCR data. An unpaired *t*-test was used for body weight and immunohistochemical analysis to detect differences between the different groups. Significance was determined at P<0.05.

## Results

### Characterization of DE

**Figure 2:**
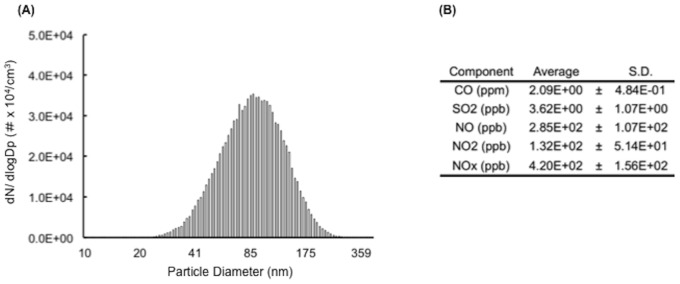
Characterization of diesel exhaust. (A) Particle diameter distribution of diesel exhaust particles. (B) Concentrations of gaseous components.

The diameter distribution of DEP in the DE chamber showed peaks at 90 nm (Figure 2A). The average number concentration of the DEP was approximately (8.1±1.0)×10^4^ (numbers/cm^3^). The average concentration of exhaust constituents was maintained at 90 µg/m^3^ [2.09 ppm for carbon monoxide (CO), 0.132 ppm for nitrogen dioxide (NO_2_), and less than 3.62×10^−3^ ppm for sulfur dioxide (SO_2_)] (Figure 2B).

### Effects of Early Environmental Enrichment on Dam and her Pups

We analyzed for FosB expression in the medial preoptic area, a part of the anterior hypothalamus of the dam at weaning. The number of FosB-positive neurons in the medial preoptic area of dam reared in environmental enrichment was significantly decreased compared with that of dam reared in a standard laboratory cage environment (Figure S2A−D). Eye opening of pups, observed at postnatal day 13 or 14, was accelerated by environmental enrichment during the perinatal period (Figure S3A). Food intake per cage (one dam and 8 pups) was significantly increased during the final lactation period (postnatal days 21−25) by environmental enrichment (Figure S3B).

### Effects of Exposure to Diesel Exhaust on the Lung

Body weight gain of control and enriched mice was similar to that of those exposed to DE (Figure S1D). We evaluated the histology of the lung of mice to confirm the induced toxicity under conditions of the present experimental design of diesel exhaust exposure. There was no remarkable difference in pathological finding among DE exposure groups (C-DE, EE-DE) and non-exposure groups (C-C, EE-C). Macrophages that phagocytized DEP were slightly observed in the bronchiolar lumen of mice after exposure to DE (C-DE, EE-DE) (Figure S4A–D).

### Profiling and Visualization of Gene Expression Pattern by cDNA Microarray and Hierarchical Clustering Analysis

**Figure 3:**
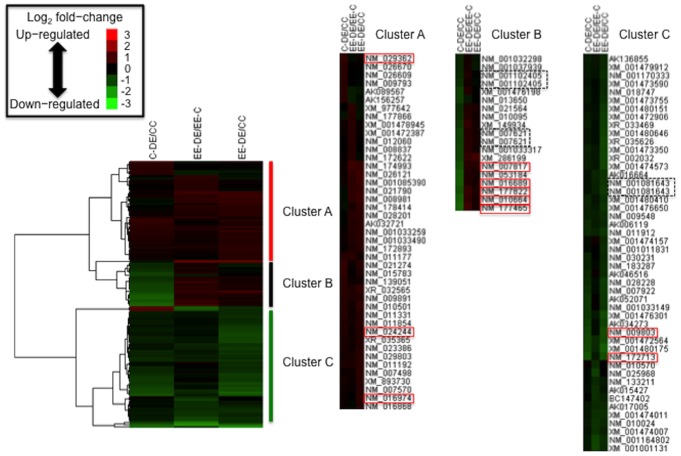
Hierarchical clustering of gene expression data. Within each group, a fold change (C-DE/C-C, EE-DE/EE-C, and EE-DE/C-C) was calculated and log_2_ transformed for all 116 spots on the microarray that did not have any missing values. These values were then hierarchically clustered using Euclidean distance metric and complete linkage. The colored images are presented as described: the color scale ranges from saturated green for log_2_ ratios –3.0 and below to saturated red for log_2_ ratios 3.0 and above. Gene expression profiles were divided into 3 clusters (clusters A, B, and C), and quantitative RT-PCR analysis was performed for genes surrounded by the red line in each cluster. Genes surrounded by black dotted line were the same gene derived from different spots.

The effects of DE exposure and rearing environment during the perinatal period on the gene expression pattern in the olfactory bulb were evaluated by microarray. From the 62,976 spots (28,620 genes) printed on the microarray, 18,190 spots (15,332 genes) were found with GenBank accession numbers and a high-quality signal. Moreover, 116 spots (112 genes) were found to be differentially expressed (1.5-fold upregulated or downregulated) either in C-DE/C-C, EE-DE/EE-C, or EE-DE/C-C comparisons (Table S1), but surprisingly, there were no gene differences between the C-C and EE-C groups. Hierarchical clustering analysis classified the 116 spots into three major clusters based on their expression patterns. We showed the combined effect of diesel exhaust exposure and rearing environment by EE-DE/C-C comparison in the heat map. The combined impact cannot be visualized by only C-DE/C-C and EE-DE/EE-C comparisons. The gene expression patterns in cluster A (43 genes) and cluster C (47 genes) were upregulated and downregulated in either C-DE/C-C or EE-DE/EE-C comparisons, respectively. The gene expression pattern in cluster B (17 genes) exhibited a different expression change between C-DE/C-C and EE-DE/EE-C comparisons. This heat map allowed us to determine how these genes were related to the effects of DE exposure with or without early environmental enrichment (Figure 3).

### Validation of Microarray Results by RT-PCR

**Figure 4:**
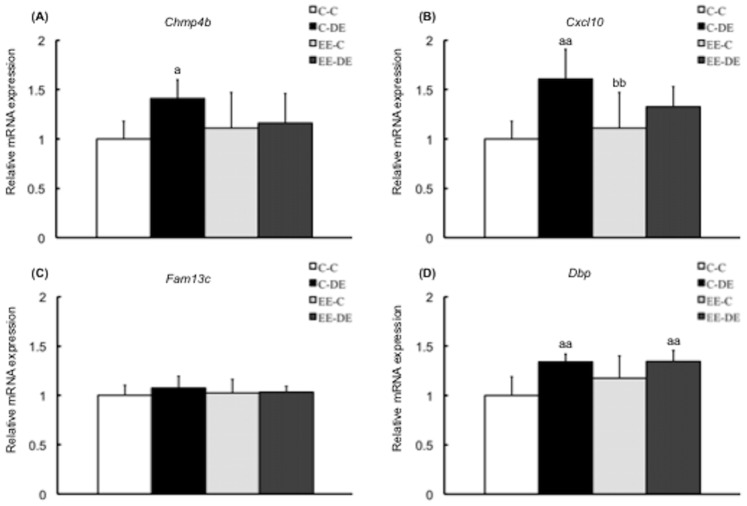
Confirmation of microarray data in cluster A by RT-PCR. Data show mRNA expressions for (A) *Chmp4b*, (B) *Cxcl10*, (C) *Fam13c*, and (D) *Dbp* in the olfactory bulb of mice in each group. *Chmp4b*, *Cxcl10*, and *Dbp*, except for *Fam13c*, of mice reared in a standard cage environment were significantly upregulated by exposure to diesel exhaust. There was no significant effect of exposure to diesel exhaust on gene expressions of mice reared in environmental enrichment. The data are expressed as relative target gene expression compared with *Gapdh* expression. Each column represents the mean ± standard deviation (C-C: n = 7, C-DE: n = 7, EE-C: n = 8, EE-DE: n = 9). Data were analyzed by two-way analysis of variance as described in the Methods section. An analysis of simple effects is as follows: *Chmp4b*: a indicates significant differences (Tukey–Kramer method, ^a^P<0.05, C-C vs. C-DE); *Cxcl10*: a indicates significant differences (Tukey–Kramer method, ^aa^P<0.01, C-C vs. C-DE); b indicates significant differences (Tukey–Kramer method, ^bb^P<0.01, C-DE vs. EE-C); and *Dbp*: a indicates significant differences (Tukey–Kramer method, ^aa^P<0.01, C-C vs. C-DE and C-C vs. EE-DE).

**Figure 5:**
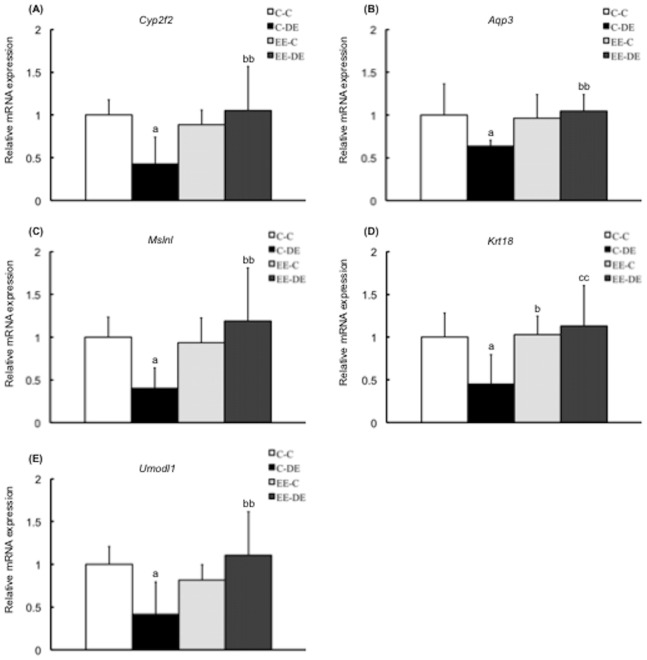
Confirmation of microarray data in cluster B by RT-PCR. Data show mRNA expressions for (A) *Cyp2f2*, (B) *Aqp3*, (C) *Mslnl*, (D) *Krt18*, and (E) *Umodl1* in the olfactory bulb of mice in each group. *Cyp2f2*, *Aqp3*, *Mslnl*, *Krt18*, and *Umodl1* of mice reared in a standard cage environment were significantly downregulated by exposure to diesel exhaust. There was no significant effect of exposure to diesel exhaust on gene expression of mice reared in environmental enrichment. The data are expressed as relative target gene expression compared with *Gapdh* expression. Each column represents the mean ± standard deviation (C-C: n = 7, C-DE: n = 7, EE-C: n = 8, EE-DE: n = 9). Data were analyzed by two-way analysis of variance as described in the Methods section. An analysis of simple effects is as follows: *Cyp2f2*: a indicates significant differences (Tukey-Kramer method, ^a^P<0.05, C-C vs. C-DE); b indicates significant differences (Tukey–Kramer method, ^bb^P<0.01, C-DE vs. EE-DE); *Aqp3*: a indicates significant differences (Tukey–Kramer method, ^a^P<0.05, C-C vs. C-DE); b indicates significant differences (Tukey-Kramer method, ^b^P<0.05, C-DE vs. EE-DE); *Mslnl*: a indicates significant differences (Tukey–Kramer method, ^a^P<0.05, C-C vs. C-DE); b indicates significant differences (Tukey–Kramer method, ^bb^P<0.01, C-DE vs. EE-DE); *Krt18*: a indicates significant differences (Tukey–Kramer method, ^a^P<0.05, C-C vs. C-DE); b indicates significant differences (Tukey-Kramer method, ^b^P<0.05, C-DE vs. EE-C); c indicates significant differences (Tukey–Kramer method, ^cc^P<0.01, C-DE vs. EE-DE); *Umodl1*: a indicates significant differences (Tukey–Kramer method, ^a^P<0.05, C-C vs. C-DE); and b indicates significant differences (Tukey–Kramer method, ^bb^P<0.01, C-DE vs. EE-DE).

**Figure 6:**
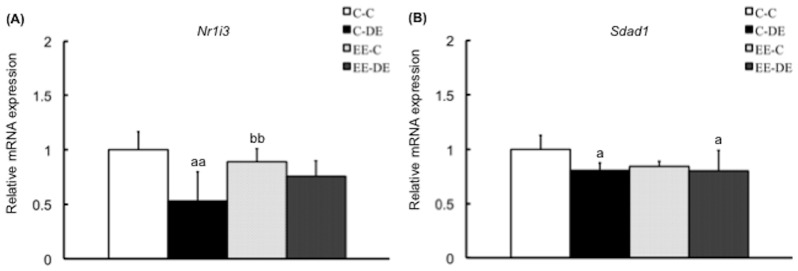
Confirmation of microarray data in cluster C by RT-PCR. Data show mRNA expressions for (A) *Nr1i3*, and (B) *Sdad1* in the olfactory bulb of mice in each group. *Nr1i3* and *Sdad1* of the olfactory bulb of mice reared in a standard cage environment were significantly downregulated by exposure to diesel exhaust. There was no significant effect of exposure to diesel exhaust on gene expressions of mice reared in environmental enrichment. The data are expressed as relative target gene expression compared with *Gapdh* expression. Each column represents the mean ± standard deviation (C-C: n = 7, C-DE: n = 7, EE-C: n = 8, EE-DE: n = 9). Data were analyzed by two-way analysis of variance as described in the Methods section. An analysis of simple effects is as follows: *Nr1i3*: a indicates significant differences (Tukey–Kramer method, ^aa^P<0.01, C-C vs. C-DE); b indicates significant differences (Tukey–Kramer method, ^bb^P<0.01, C-DE vs. EE-C); and *Sdad1*: a indicates significant differences (Tukey–Kramer method, ^a^P<0.05, C-C vs. C-DE and C-C vs. EE-DE).

We conducted RT-PCR quantification of the expression of 11 selected genes in a second set of samples (not included in the microarray experiments) to validate the microarray data and obtain expression data for each sample. All PCR reactions had efficiencies greater than 90%. From the 11 selected genes, except for *Fam13c*, RT-PCR analysis validated the results for 10 genes: *Chmp4b*, *Cxcl10* and *Dbp* in cluster A (Figure 4A–D), *Cyp2f2*, *Aqp3*, *Mslnl*, *Krt18* and *Umodl1* in cluster B (Figure 5A–E), and *Nr1i3* and *Sdad1* in cluster C (Figure 6A, B). Interestingly, there was no difference in gene expression levels between EE-C and EE-DE, whereas expression levels of the genes were dysregulated by DE exposure of mice reared in a standard cage environment. As shown in Figure 4, for *Chmp4b*, two-way ANOVA showed significant main effect for DE exposure [F (1, 27) = 4.57, P<0.05] without DE exposure/rearing environment interaction; for *Cxcl10*, two-way ANOVA showed significant main effect for DE exposure [F (1, 27) = 16.34, P<0.001] without DE exposure/rearing environment interaction; for *Fam13c*, two-way ANOVA failed to find a significant main effect of DE exposure; and for *Dbp*, two-way ANOVA showed significant main effect for DE exposure [F (1, 27) = 18.35, P<0.001] without DE exposure/rearing environment interaction. As shown in Figure 5, for *Cyp2f2*, two-way ANOVA showed significant main effect for rearing environment [F (1, 27) = 4.73, P<0.001] with significant DE exposure/rearing environment interaction [F (1, 27) = 9.19, P<0.01]; for *Aqp3*, two-way ANOVA showed significant main effect for rearing environment [F (1, 27) = 4.58, P<0.05] with significant DE exposure/rearing environment interaction [F (1, 27) = 6.23, P<0.05]; for *Mslnl*, two-way ANOVA showed significant main effect for rearing environment [F (1, 27) = 6.61, P<0.05] with significant DE exposure/rearing environment interaction [F (1, 27) = 8.67, P<0.01]; for *Krt18*, two-way ANOVA showed no significant main effect for DE exposure and rearing environment with significant DE exposure/rearing environment interaction [F (1, 27) = 8.39, P<0.01]; and for *Umodl1*, two-way ANOVA showed no significant main effect for DE exposure and rearing environment with significant DE exposure/rearing environment interaction [F (1, 27) = 11.37, P<0.01]. As shown in Figure 6, for *Nr1i3*, two-way ANOVA showed significant main effect for DE exposure [F (1, 27) = 19.87, P<0.001] with significant DE exposure/rearing environment interaction [F (1, 27) = 6.93, P<0.05], and for *Sdad1*, two-way ANOVA showed significant main effect for DE exposure [F (1, 27) = 6.05, P<0.05] without a DE exposure/rearing environment interaction.

### Functional Classification of Microarray Data

Functional analysis using GO revealed that 112 genes (on the 116 spots) were enriched in nine potentially important categories with both a high enrichment factor (≥5) and statistical significance (P<0.05). The largest group of the functional categories was those related to immune and inflammation modulation. In particular, “inflammatory response” and “innate immune response” were included in the largest number of dysregulated genes (five genes), together with “immune response” (four genes), “chemotaxis” (three genes), and “chemokine activity” (two genes). Other interesting categories were “defense response to virus” (five genes), “response to virus” (three genes), and “sensory perception of smell” (three genes) (Table 2). Six pathways were also identified from the 112 genes data (Table 3). Biological pathway analysis incriminated related pathways, which were modulated by DE exposure. Several immune-related pathways, including “RIG-I-like receptor signaling pathway”, “Toll-like receptor signaling pathway”, “Chemokine receptors bind chemokines”, “Interferon alpha beta signaling”, “Toll pathway”, and “IL4 pathway” were significantly overrepresented. The results of the GO analysis showed that the genes in clusters A, B and C were enriched in eight (Table 4), three (Table 5) and one (Table 6) potentially important categories, respectively. Six genes in cluster A, three genes in cluster B, and 15 genes in cluster C were not annotated to any GO.

**Table 2:** Functional analysis of microarray data using Gene Ontology (GO).

GO		Nf	P value	Enrichment factor	GenBank Accession	Gene symbol
ID	Term					
GO:0051607	defense response to virus	62	<0.001	11.41	NM_021274	Cxcl10
					NM_010501	Ifit3
					NM_011854	Oasl2
					NM_133211	Tlr7
					NM_015783	Isg15
GO:0006954	inflammatory response	94	<0.001	7.52	NM_009793	Camk4
					NM_021274	Cxcl10
					NM_013650	S100a8
					NM_011331	Ccl12
					NM_133211	Tlr7
GO:0045087	innate immune response	97	<0.001	7.29	NM_010501	Ifit3
					NM_013650	S100a8
					NM_011854	Oasl2
					NM_001170333	Clec4a2
					NM_133211	Tlr7
GO:0006955	immune response	62	<0.001	9.13	NM_021274	Cxcl10
					NM_011331	Ccl12
					NM_011854	Oasl2
					NM_012060	Bcap31
GO:0006935	chemotaxis	41	<0.01	10.35	NM_021274	Cxcl10
					NM_013650	S100a8
					NM_011331	Ccl12
GO:0009615	response to virus	32	<0.01	13.26	NM_010501	Ifit3
					NM_011854	Oasl2
					NM_029803	Ifi27l2a
GO:0007608	sensory perception of smell	31	<0.01	13.69	NM_053184	Ugt2a1
					NM_001033317	Cnga4
					NM_001011831	Olfr1500
GO:0008009	chemokine activity	14	<0.01	20.21	NM_021274	Cxcl10
					NM_011331	Ccl12

**Table 3:** Summary of gene set enrichment analysis.

Pathway	Nf	P value	Enrichment factor	GenBank Accession	Gene symbol
RIG I LIKE RECEPTOR SIGNALING PATHWAY (KEGG)	38	<0.05	8.37	NM_021274	Cxcl10
				NM_015783	Isg15
TOLL LIKE RECEPTOR SIGNALING PATHWAY (KEGG)	49	<0.05	6.49	NM_021274	Cxcl10
				NM_133211	Tlr7
CHEMOKINE RECEPTORS BIND CHEMOKINES (REACTOME)	14	<0.01	22.72	NM_021274	Cxcl10
				NM_011331	Ccl12
INTERFERON ALPHA BETA SIGNALING (REACTOME)	39	<0.05	8.16	NM_010501	Ifit3
				NM_015783	Isg15
TOLL PATHWAY (BIOCARTA)	26	<0.05	12.23	NM_007922	Elk1
				NM_133211	Tlr7
IL4 PATHWAY (BIOCARTA)	7	<0.05	22.72	NM_010570	Irs1

Gene set enrichment analysis of the microarray results for diesel exhaust exposure vs. control identified gene sets correlated with inflammatory and immune systems. *Cxcl10* expression by microarray was confirmed by quantitative RT-PCR.

**Table 4:** Functional analysis of cluster A using Gene Ontology (GO).

GO		Nf	P value	Enrichment factor	GenBank Accession	Gene symbol
ID	Term					
GO:0051607	defense response to virus	62	<0.001	21.21	NM_021274	Cxcl10
					NM_010501	Ifit3
					NM_011854	Oasl2
					NM_015783	Isg15
GO:0006955	immune response	62	<0.001	21.21	NM_021274	Cxcl10
					NM_011331	Ccl12
					NM_011854	Oasl2
					NM_012060	Bcap31
GO:0009615	response to virus	32	<0.001	30.82	NM_010501	Ifit3
					NM_011854	Oasl2
					NM_029803	Ifi27l2a
GO:0006954	inflammatory response	94	<0.01	10.49	NM_009793	Camk4
					NM_021274	Cxcl10
					NM_011331	Ccl12
GO:0008009	chemokine activity	14	<0.001	46.97	NM_021274	Cxcl10
					NM_011331	Ccl12
GO:0006935	chemotaxis	41	<0.01	16.04	NM_021274	Cxcl10
					NM_011331	Ccl12
GO:0005125	cytokine activity	58	<0.05	11.34	NM_021274	Cxcl10
					NM_011331	Ccl12
GO:0045087	innate immune response	97	<0.05	6.78	NM_010501	Ifit3
					NM_011854	Oasl2

The results of the gene annotation of cluster A using GO identified gene sets correlated with inflammatory and immune systems. *Cxcl10* expression by microarray was confirmed by quantitative RT-PCR.

**Table 5:** Functional analysis of cluster B using Gene Ontology (GO).

GO		Nf	P value	Enrichment factor	GenBank Accession	Gene symbol
ID	Term					
GO:0005576	extracellular region	625	<0.001	6.95	NM_001037939	Bglap
					NM_001032298	Bglap2
					NM_013650	S100a8
					NM_177465	Umodl1
					NM_021564	Fetub
GO:0005615	extracellular space	335	<0.001	12.97	NM_001102405	Acp5
					NM_001032298	Bglap2
					NM_013650	S100a8
					NM_177465	Umodl1
					NM_021564	Fetub
GO:0005509	calcium ion binding	343	<0.001	10.13	NM_001037939	Bglap
					NM_001032298	Bglap2
					NM_013650	S100a8
					NM_177465	Umodl1

The results of the gene annotation of cluster B using GO identified gene sets correlated with abovementioned GO terms. *Umodl1* expression by microarray was confirmed by quantitative RT-PCR.

**Table 6:** Functional analysis of cluster C using Gene Ontology (GO).

GO		Nf	P value	Enrichment factor	GenBank Accession	Gene symbol
ID	Term					
GO:0045087	innate immune response	97	<0.05	7.84	NM_001170333	Clec4a2
					NM_133211	Tlr7

The results of the gene annotation of cluster C using GO identified gene sets correlated with inflammatory and immune systems.

## Discussion

There is increasing evidence that DE inhalation may result in neurotoxicity [13], [14], [34], but the effect of a low concentration of subacute DE exposure on the central nervous system is poorly understood. The present study provides experimental evidence that 28-day DE exposure at an environmental concentration dysregulated gene expression in the olfactory bulb of mice reared in a standard cage environment. In addition, we demonstrated that environmental enrichment during the perinatal period reversed the results of gene expression in the olfactory bulb induced by DE inhalation. The results of the present study indicate for the first time that the effect of DE exposure on gene expression patterns was influenced by rearing environment during the perinatal period.

First, we characterized particle size distribution and mass concentration of DEP. We produced a mass concentration of DEP at 90 µg/m^3^, which is environmentally relevant. This approach using a DE inhalation chamber is more relevant to human exposure scenarios than other methods of exposure, such as nasal drop, oral or intratracheal DEP administration. There is an emerging concern about the effects of suspended PM in air pollutants mainly derived from DEP [6]. Numerous urban areas in the world demonstrate PM concentrations of 200 to 600 µg/m^3^ in annual averages and frequently exceeding a peak concentration of 1,000 µg/m^3^ [35]. Under the worst conditions in the United States and assuming a ventilation rate of 6 L/minute (8.6 m^3^/day) for a healthy adult at rest, the total amount of PM exposure would be 4,600 µg. This would correspond to approximately 40 µg/day of PM exposure for a mouse with a ventilation rate of 35–50 mL/minute [36]. The DE exposure in this study was approximately 2.2 µg/day. In the present study, the DE exposure condition for DEP mass concentration and exposure time was designed to be lower than comparative recent experimental studies on the effect of inhalation exposure to DE on the central nervous system [13], [14], [37], [38], [39], [40].

Our microarray data showed that exposure to a low concentration of DE changed expression of 112 genes in the olfactory bulb of mice (Table S1). Moreover, quantitative RT-PCR analysis confirmed 10 genes from the microarray data. The results suggested that two replicates of the pooled sample detected unreliable data with relatively low signal intensity and improved measurement precision. It was reported that fold change analysis alone is an unreliable indicator [41], [42]. Several publications have made specific recommendations on the number of replicates required to detect dysregulated genes based on fold change criteria [43], [44]. The major advantage of this approach was that averaging across replicates increased the precision of gene expression measurements and allowed smaller changes to be detected. In fact, in the present study, the quantitative RT-PCR analysis results supported the accuracy of the microarray data.

Second, we investigated by cDNA microarray how rearing environment during the perinatal period altered the effects of DE exposure on gene expression in the olfactory bulb. GO and pathway analysis were conducted to obtain biological and functional analysis from the microarray data. GO terms and pathways related to immune and inflammation responses were largely extracted from the data of 112 genes. These results suggested that a low concentration exposure to DE affected the expression of genes involved in immune and inflammatory responses in the olfactory bulb. These results are consistent with data from previous studies indicating that DE and DEP triggered oxidative stress and inflammation in brain tissue [12], [13], [14], [45].

To further investigate the biological and functional meanings of microarray data, we conducted hierarchical clustering to classify gene expression patterns in each group. The gene expression patterns in cluster A and cluster C were upregulated and downregulated in either C-DE/C-C or EE-DE/EE-C comparisons, respectively. In addition, quantitative RT-PCR analysis revealed a main effect of exposure to DE on the expression of genes in cluster A and cluster C without a DE exposure/environmental enrichment interaction. These results suggested that changes in the gene expression pattern in cluster A and cluster C could be attributed to the effect of DE exposure. We also demonstrated that functional analysis of cluster A using gene annotation with GO obtained a higher enrichment factor of each category involved in immune/inflammatory responses and response to virus than that of all dysregulated genes. This observation suggested that cluster A functionalized categories related to immune/inflammatory responses and response to virus compared with the genes dysregulated by DE exposure. In contrast, a subset of genes in cluster B exhibited a different expression change between C-DE/C-C and EE-DE/EE-C comparisons. In addition, quantitative RT-PCR analysis revealed a main effect of rearing environment on gene expression in cluster B with a significant DE exposure/rearing environment interaction. These results suggested that cluster B could be associated with an interaction between early rearing environment and DE exposure. Cluster B contained categories that did not include genes to immune and inflammatory responses. These results suggested that cluster B might enrich some new functional outputs by both early rearing environment and DE exposure. Therefore, the present study suggested that categorizing patterns of gene expression could identify multiple factors contributing to changes in gene expression patterns. In addition, 15 genes, such as *Sdad1*, were not annotated to any GO in cluster C. Hierarchical cluster analysis categorized actual gene expression patterns, which could be used to infer functional roles for unknown genes in cluster C. However, it is notable that cluster analysis does not give absolute answers, although there are data-mining techniques that allow relationships in the data to be explored. Further investigation will be needed to investigate a function and localization *in situ* of genes that were not annotated to any GO.

Finally, quantitative RT-PCR analysis revealed that DE exposure dysregulated gene expression levels in the olfactory bulb of mice reared in a standard cage environment, whereas no difference between control and DE-exposed mice reared in conditions of environmental enrichment was detected (Figures 4, 5, 6). These results suggested that early rearing environment might influence the effects of DE exposure. Early rearing environment, in particular, mother-infant interaction is essential for brain development. The medial preoptic area of the hypothalamus is a critical region for the expression of maternal behavior in rodents, and neurons in the medial preoptic area are active during maternal behavior as demonstrated by immunohistochemical analysis of FosB [46], [47]. Expression of FosB in the medial preoptic area of dams has been shown to be necessary for nurturing [48], [49]. The present study showed that expression levels of FosB in the medial preoptic area of dams reared in environmental enrichment significantly decreased compared with that of dams reared in a standard cage environment at weaning (Figure S2A−D). In addition, the present study also showed that pups reared in environmental enrichment during the perinatal period accelerated eye opening (Figure S3A) and increased feeding at postnatal days 21–25 (Figure S3B), suggesting that early environmental enrichment could promote pup development because of possible changes in the interactions between mother and pups, which was consistent with results from a previous study [50]. Perinatal environmental stress can have persistent effects on the mother, which may influence maternal behavior. In rodents, repeated daily restraint, lack of environmental enrichment, during the perinatal period decreased maternal care to the pups [51]. Because adoption reverses the negative effects of perinatal stress on hypothalamic-pituitary-adrenal axis function, it was hypothesized that disturbance of the mother-infant interaction may cause stress on the offspring and contribute to the long-term effects of perinatal stress. Similar mechanisms are suspected for the immune alterations observed in perinatally stressed animals [52]. Mother–infant interactions were also regulated by the olfactory system [53]. It has been reported that early environmental stress affected the magnitude of maternal behavior and nest odor preference modulated by the olfactory bulb in pups [54]. Aggressive behavior and olfactory bulb structure were altered by the laboratory cage environment [55]. Dysfunction of the olfactory bulb leads to numerous immune changes, such as reduced neutrophil phagocytosis, lymphocyte mitogenesis, lymphocyte number and negative acute phase proteins, increased leukocyte adhesiveness/aggregation, monocyte phagocytosis, neutrophil number and positive acute phase proteins [56]. It has been reported that activation of the inflammatory system in olfactory dysfunction changes immune response to further immune challenges [57]. However, little is known whether early rearing environment exacerbates the effects of exposure to DE on the central nervous system. Critical developmental windows of vulnerability of the immune system to environmental programming are presently unknown, but the present study showed that effects of exposure to DE on gene expression involved in the immune system in the olfactory bulb were dependent on the rearing environment.

Previous studies have reported the early environmental origins of neurodegenerative disease in later life [58]. Mice housed in a standard laboratory cage setting exhibited higher rates of stereotypical behaviors at early development [15] than those of mice in environmental enrichment conditions [17]. These findings suggest the importance of environmental factors, such as rearing environment during the perinatal period in investigational neurotoxicology studies. We focused on the olfactory bulb because the olfactory bulb is important for the maintenance of psychiatric illnesses, such as depression [57]. The present study showed for the first time that rearing environment during the perinatal period influenced the effects of DE exposure on the olfactory bulb. The International Agency for Research on Cancer concluded that DE is carcinogenic to humans (Group 1) in 2012, although recently DEP concentrations in the environment have been significantly decreased. However, there is little evidence of the effects of exposure to DE at environmental concentrations on the central nervous system. We have just begun to observe the effect of low concentrations of DE on the central nervous system. Our findings suggest that the influence on the developing brain of housing environment, such as environmental enrichment in early life, might be an important contributor to the effects of previously unidentified toxic environmental agents, such as DE.

In conclusion, our study demonstrates that DE-induced dysregulated genes of the olfactory bulb were influenced by early housing environment. We reported that 28-day DE exposure affected immune and inflammatory responses when reared in a standard cage environment during the perinatal period, but not when reared in environmental enrichment during this same period. These results provide novel insights regarding housing environment for the evaluation of health effects of DE. Further investigation is required to investigate the precise mechanisms of immune response and histological analyses of environmental concentrations of DE on olfactory bulb of mice reared in different housing environments.

## Supporting Information

Figure S1 Body weight. There was no difference in body weight between mice in control and environmental enrichment groups (Unpaired *t*-test). The data are expressed as a mean of the value of body weight in the control dam and environmental enrichment dam at (A) gestational day 14 (n = 9) and (B) weaning (n = 9). The data are expressed as a mean of the value of body weight (C) in control pups and environmental enrichment pups at postnatal (P) days 10 and 26 (n = 10). (D) The data are expressed as a mean of the value of the changed body weight in male offspring by 28-day diesel exhaust inhalation (C-C: n = 7, C-DE: n = 7, EE-C: n = 8, EE-DE: n = 9). Each column represents the mean ± standard deviation.
(TIFF)

Figure S2 Data by immunohistochemical analysis show FosB expression in the medial preoptic area of the hypothalamus of dam at weaning. Images show a representation of the immunostaining procedure that labeled FosB as black [(A) Control, (B) Environmental enrichment]. Scale bar = 100 µm. (C) Mean (± standard deviation) numbers of FosB-positive cells in the medial preoptic area of the hypothalamus: flesh color indicated in (D) of control and environmental enrichment dam (n = 3). Environmental enrichment decreased FosB-positive cells in the medial preoptic area of the hypothalamus (Unpaired *t*-test, **P<0.01).
(TIFF)

Figure S3 Effect of environmental enrichment during the perinatal period on pup development. (A) Environmental enrichment during the perinatal period increased food intake. A dam and her pups (n = 7–9 per cage) in a home cage were considered to be one cage (N = 1). The graph shows the mean food intake of white circles (control cage; N = 8) and black squares (environmental enrichment cage; N = 9) for each age. On postnatal (P) days 21–25, environmental enrichment increased food intake (Tukey–Kramer method, **P<0.01.). Values are mean ± standard deviation. (B) Precocious eye opening in environmentally enriched mice. The percentage of postnatal (P) day 13 (white column) and P 14 (black column) pups that opened their eyes in the indicated cages is shown.
(TIFF)

Figure S4 Effect of exposure to diesel exhaust on lung tissue. Images show a representation of histology of lung by exposure to clean air [(A): C-C, (C): EE-C] or diesel exhaust [(B): C-DE, (D): EE-DE]. Scale bar = 20 µm. (B, D) Macrophages that phagocytized diesel exhaust particles were observed in the bronchiolar lumen of mice (arrow). However, there was no difference in pathological findings among groups.
(TIFF)

Table S1 List of significantly expressed genes.
(DOC)
