# Supplementary figures and images for: Gene Expression Changes in the Olfactory Bulb of Mice Induced by Exposure to Diesel Exhaust Are Dependent on Animal Rearing Environment
Source: PLoS One. 2013 Aug 5;8(8):e70145. doi: 10.1371/journal.pone.0070145 (PMC3734019; doi:10.1371/journal.pone.0070145)

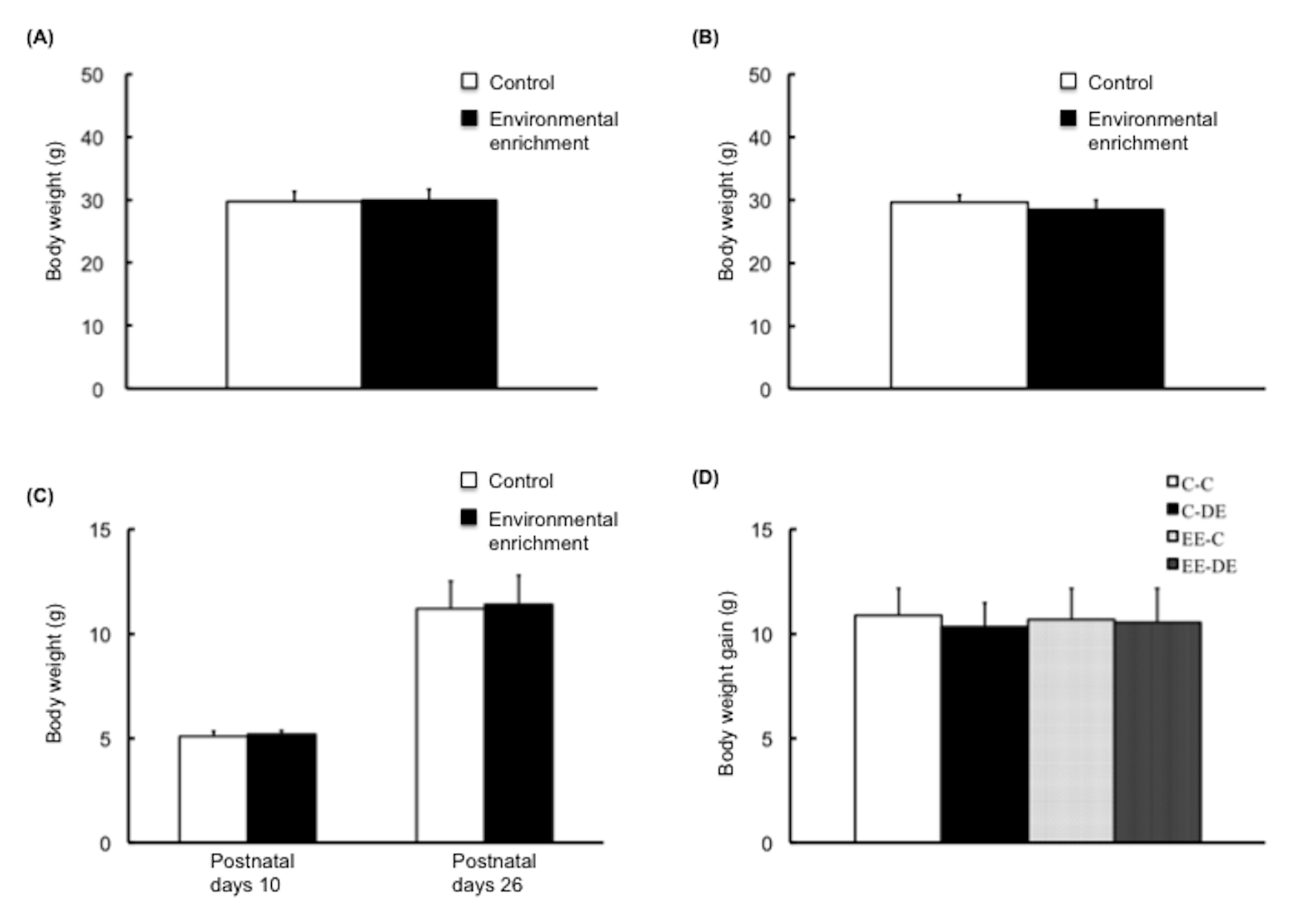

Supplement: Figure S1 — Body weight. There was no difference in body weight between mice in control and environmental enrichment groups (Unpaired t-test). The data are expressed as a mean of the value of body weight in the control dam and environmental enrichment dam at (A) gestational day 14 (n = 9) and (B) weaning (n = 9). The data are expressed as a mean of the value of body weight (C) in control pups and environmental enrichment pups at postnatal (P) days 10 and 26 (n = 10). (D) The data are expressed as a mean of the value of the changed body weight in male offspring by 28-day diesel exhaust inhalation (C-C: n = 7, C-DE: n = 7, EE-C: n = 8, EE-DE: n = 9). Each column represents the mean ± standard deviation. (TIFF) [file pone.0070145.s001.tiff]

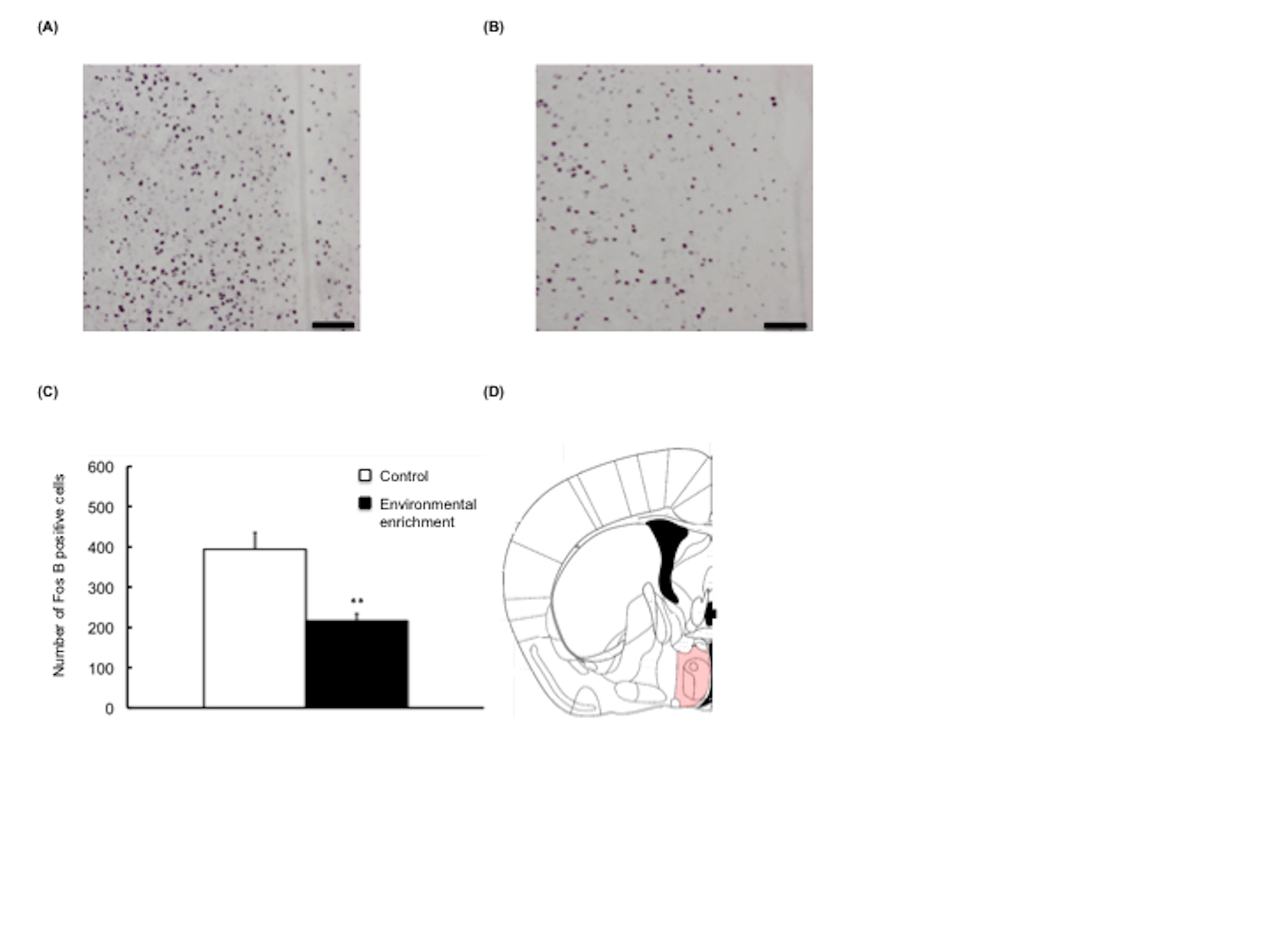

Supplement: Figure S2 — Data by immunohistochemical analysis show FosB expression in the medial preoptic area of the hypothalamus of dam at weaning. Images show a representation of the immunostaining procedure that labeled FosB as black [(A) Control, (B) Environmental enrichment]. Scale bar = 100 µm. (C) Mean (± standard deviation) numbers of FosB-positive cells in the medial preoptic area of the hypothalamus: flesh color indicated in (D) of control and environmental enrichment dam (n = 3). Environmental enrichment decreased FosB-positive cells in the medial preoptic area of the hypothalamus (Unpaired t-test, **P<0.01). (TIFF) [file pone.0070145.s002.tiff]

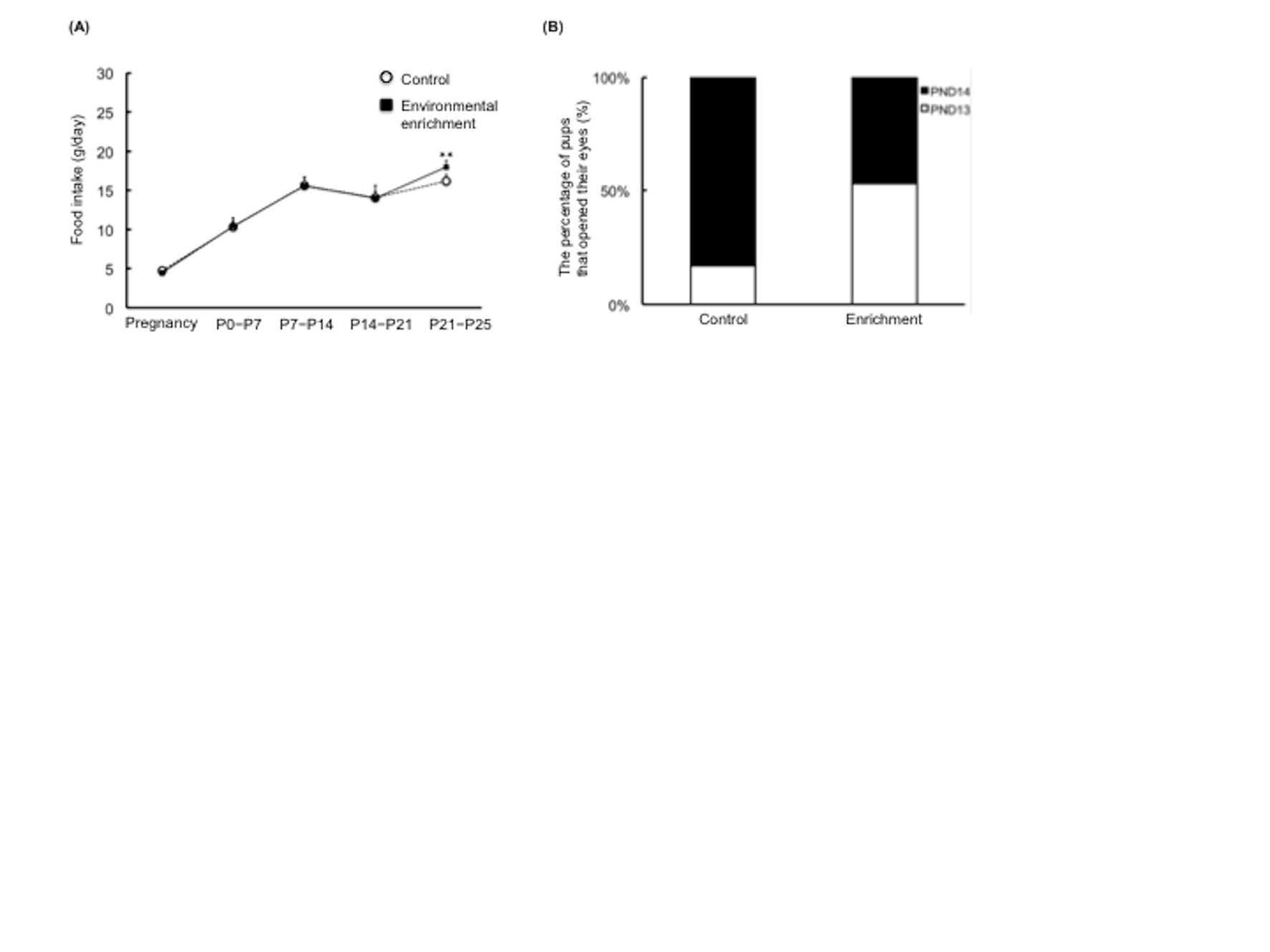

Supplement: Figure S3 — Effect of environmental enrichment during the perinatal period on pup development. (A) Environmental enrichment during the perinatal period increased food intake. A dam and her pups (n = 7–9 per cage) in a home cage were considered to be one cage (N = 1). The graph shows the mean food intake of white circles (control cage; N = 8) and black squares (environmental enrichment cage; N = 9) for each age. On postnatal (P) days 21–25, environmental enrichment increased food intake (Tukey–Kramer method, **P<0.01.). Values are mean ± standard deviation. (B) Precocious eye opening in environmentally enriched mice. The percentage of postnatal (P) day 13 (white column) and P 14 (black column) pups that opened their eyes in the indicated cages is shown. (TIFF) [file pone.0070145.s003.tiff]

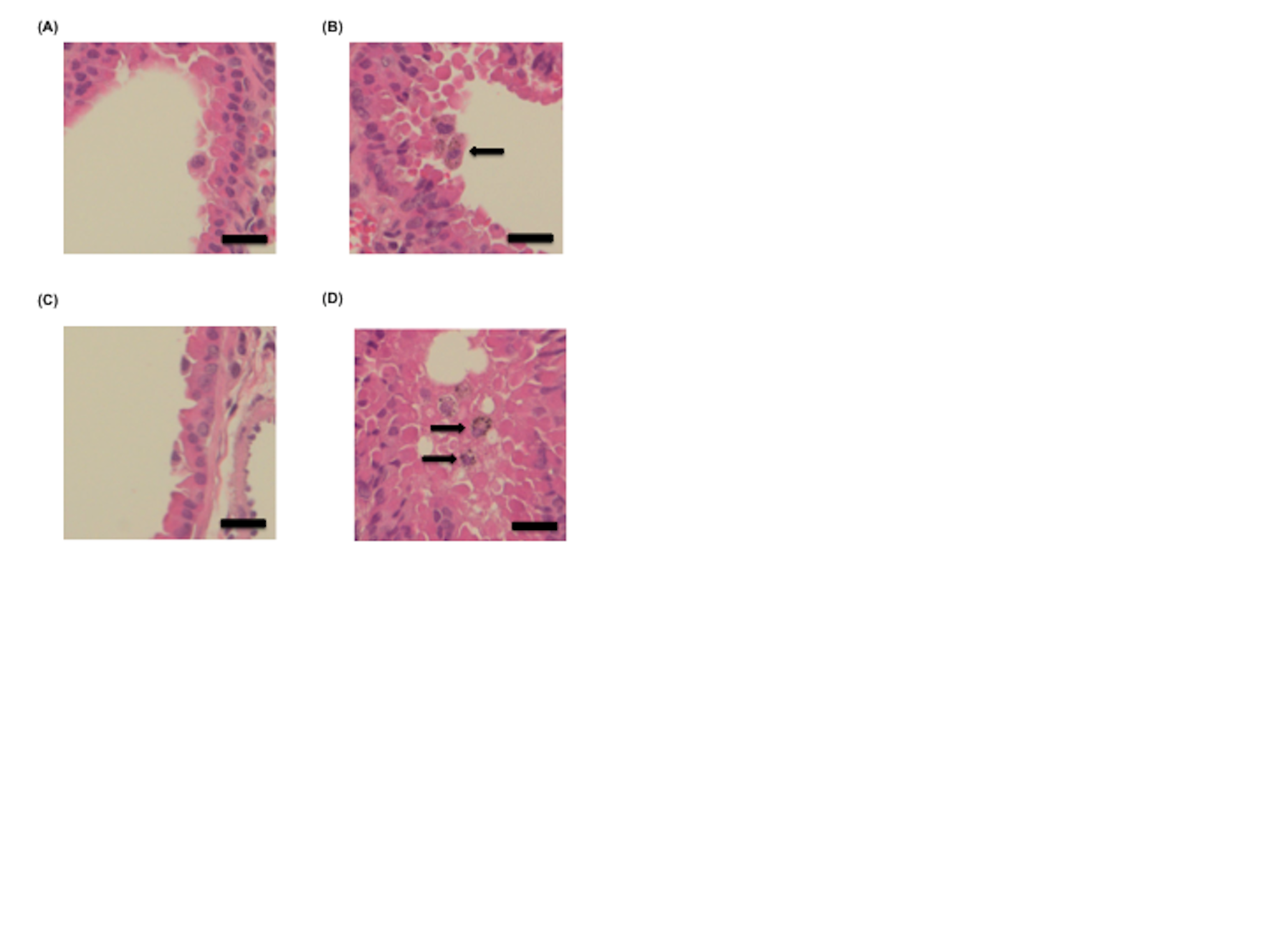

Supplement: Figure S4 — Effect of exposure to diesel exhaust on lung tissue. Images show a representation of histology of lung by exposure to clean air [(A): C-C, (C): EE-C] or diesel exhaust [(B): C-DE, (D): EE-DE]. Scale bar = 20 µm. (B, D) Macrophages that phagocytized diesel exhaust particles were observed in the bronchiolar lumen of mice (arrow). However, there was no difference in pathological findings among groups. (TIFF) [file pone.0070145.s004.tiff]
