# Supplementary material for: Gene Expression Changes in the Olfactory Bulb of Mice Induced by Exposure to Diesel Exhaust Are Dependent on Animal Rearing Environment
Source: PLoS One. 2013 Aug 5;8(8):e70145. doi: 10.1371/journal.pone.0070145 (PMC3734019; doi:10.1371/journal.pone.0070145)
Supplement: Table S1 — List of significantly expressed genes. (DOC) [file pone.0070145.s005.doc]

Table S1. List of significantly expressed genes.

| GenBank Accession | Description | Gene Symbol |
| --- | --- | --- |
|
| NM_029362 | chromatin modifying protein 4B | *Chmp4b* |
| NM_026670 | zinc finger, MYM domain containing 1 | *Zmym1* |
| NM_026609 | leptin receptor overlapping transcript-like 1 | *Leprotl1* |
| NM_009793 | calcium/calmodulin-dependent protein kinase IV | *Camk4* |
| AK089567 | ― | ― |
| AK156257 | ― | ― |
| XM_977642 | predicted gene 7677 | *Gm7677* |
| NM_177866 | cation channel, sperm associated 4 | *Catsper4* |
| XM_001478945 | predicted gene 4077 | *Gm4077* |
| XM_001472387 | similar to Ubtf protein | *LOC100044473* |
| NM_012060 | B-cell receptor-associated protein 31 | *Bcap31* |
| NM_008837 | protein interacting with C kinase 1 | *Pick1* |
| NM_172622 | transcriptional regulating factor 1 | *Trerf1* |
| NM_174993 | fragile X mental retardation 1 neighbor | *Fmr1nb* |
| NM_026121 | BCL2-associated athanogene 4 | *Bag4* |
| NM_001085390 | dual specificity phosphatase 5 | *Dusp5* |
| NM_021790 | centromere protein K | *Cenpk* |
| NM_008981 | protein tyrosine phosphatase, receptor type, G | *Ptprg* |
| NM_178414 | acyl-CoA synthetase medium-chain family member 4 | *Acsm4* |
| NM_028201 | RIKEN cDNA 2210009G21 gene | *2210009G21Rik* |
| AK032721 | ― | ― |
| NM_001033259 | coiled-coil domain containing 109A | *Ccdc109a* |
| NM_001033490 | pseudouridylate synthase-like 1 | *Pusl1* |
| NM_172893 | poly (ADP-ribose) polymerase family, member 12 | *Parp12* |
| NM_011177 | kallikrein related-peptidase 6 | *Klk6* |
| NM_021274 | chemokine (C-X-C motif) ligand 10 | *Cxcl10* |
| NM_015783 | ISG15 ubiquitin-like modifier | *Isg15* |
| NM_139051 | nuclear receptor subfamily 5, group A, member 1 | *Nr5a1* |
| XR_032565 | predicted gene 8985 | *Gm8985* |
| NM_009891 | choline acetyltransferase | *Chat* |
| NM_010501 | interferon-induced protein with tetratricopeptide repeats 3 | *Ifit3* |
| NM_011331 | chemokine (C-C motif) ligand 12 | *Ccl12* |
| NM_011854 | 2'-5' oligoadenylate synthetase-like 2 | *Oasl2* |
| NM_024244 | family with sequence similarity 13, member C | *Fam13c* |
| XR_035365 | ― | ― |
| NM_023386 | receptor transporter protein 4 | *Rtp4* |
| NM_029803 | interferon, alpha-inducible protein 27 like 2A | *Ifi27l2a* |
| NM_011192 | proteaseome (prosome, macropain) 28 subunit, 3 | *Psme3* |
| NM_007498 | activating transcription factor 3 | *Atf3* |
| XM_893730 | predicted gene 6934 | *Gm6934* |
| NM_007570 | B-cell translocation gene 2, anti-proliferative | *Btg2* |
| NM_016974 | D site albumin promoter binding protein | *Dbp* |
| NM_016868 | hypoxia inducible factor 3, alpha subunit | *Hif3a* |
| NM_199366 | galactose-3-O-sulfotransferase 2 | *Gal3st2* |
| NM_001032298 | bone gamma-carboxyglutamate protein 2 | *Bglap2* |
| NM_001037939 | bone gamma carboxyglutamate protein | *Bglap* |
| NM_001102405 | acid phosphatase 5, tartrate resistant | *Acp5* |
| NM_001102405 | acid phosphatase 5, tartrate resistant | *Acp5* |
| XM_001476198 | ― | ― |
| NM_013650 | S100 calcium binding protein A8 (calgranulin A) | *S100a8* |
| NM_021564 | fetuin beta | *Fetub* |
| NM_010095 | early B-cell factor 2 | *Ebf2* |
| XM_149934 | predicted gene 581 | *Gm581* |
| NM_007621 | carbonyl reductase 2 | *Cbr2* |
| NM_007621 | carbonyl reductase 2 | *Cbr2* |
| NM_001033317 | cyclic nucleotide gated channel alpha 4 | *Cnga4* |
| XM_286199 | cytochrome P450, family 2, subfamily a, polypeptide 21, pseudogene | *Cyp2a21-ps* |
|
| NM_007817 | cytochrome P450, family 2, subfamily f, polypeptide 2 | *Cyp2f2* |
| NM_053184 | UDP glucuronosyltransferase 2 family, polypeptide A1 | *Ugt2a1* |
| NM_016689 | aquaporin 3 | *Aqp3* |
| NM_177822 | mesothelin-like | *Mslnl* |
| NM_010664 | keratin 18 | *Krt18* |
| NM_177465 | uromodulin-like 1 | *Umodl1* |
| AK172117 | ― | ― |
| AK089567 | ― | ― |
| AK136855 | RIKEN cDNA A530083I20 gene | *A530083I20Rik* |
| XM_001479912 | predicted gene 9444 | *Gm9444* |
| NM_001170333 | C-type lectin domain family 4, member a2 | *Clec4a2* |
| XM_001473590 | predicted gene 2437 | *Gm2437* |
| NM_018747 | A kinase (PRKA) anchor protein 7 | *Akap7* |
| XM_001473755 | predicted gene 2488 | *Gm2488* |
| XM_001480151 | predicted gene 4429 | *Gm4429* |
| XM_001472906 | hypothetical protein LOC100043976 | *LOC100043976* |
| XR_033469 | similar to Interferon-activatable protein 203 (Ifi-203) (Interferon-inducible protein p203) | *LOC677308* |
|
| XM_001480646 | predicted gene 4578 | *Gm4578* |
| XR_035626 | predicted gene, EG668249 | *EG668249* |
| XM_001473350 | similar to Ubtf protein | *LOC100044930* |
| XR_002032 | predicted gene 8659 | *Gm8659* |
| XM_001474573 | predicted gene, EG622110 | *EG622110* |
| AK016664 | RIKEN cDNA 4933405E24 gene | *4933405E24Rik* |
| NM_001081643 | X-linked lymphocyte-regulated 3B | *Xlr3b* |
| NM_001081643 | X-linked lymphocyte-regulated 3B | *Xlr3b* |
| XM_001480410 | predicted gene 4522 | *Gm4522* |
| XM_001476650 | predicted gene 9147 | *Gm9147* |
| NM_009548 | ring finger protein 112 | *Rnf112* |
| AK006119 | ― | ― |
| NM_011912 | ventral anterior homeobox containing gene 2 | *Vax2* |
| XM_001474157 | hypothetical protein LOC100043999 | *LOC100043999* |
| NM_001011831 | olfactory receptor 1500 | *Olfr1500* |
| NM_030231 | ATP/GTP binding protein-like 4 | *Agbl4* |
| NM_183287 | RIKEN cDNA 2610318N02 gene | *2610318N02Rik* |
| AK046516 | ― | ― |
| NM_028228 | PIN2/TERF1 interacting, telomerase inhibitor 1 | *Pinx1* |
| NM_007922 | ELK1, member of ETS oncogene family | *Elk1* |
| AK052071 | ― | ― |
| NM_001033149 | tetratricopeptide repeat domain 9 | *Ttc9* |
| XM_001476301 | predicted gene 3306 | *Gm3306* |
| AK034273 | ― | ― |
| NM_009803 | nuclear receptor subfamily 1, group I, member 3 | *Nr1i3* |
| XM_001472564 | similar to Adam6 protein | *LOC100044553* |
| XM_001480175 | predicted gene 4440 | *Gm4440* |
| NM_172713 | SDA1 domain containing 1 | *Sdad1* |
| NM_010570 | insulin receptor substrate 1 | *Irs1* |
| NM_025968 | prostaglandin reductase 1 | *Ptgr1* |
| NM_133211 | toll-like receptor 7 | *Tlr7* |
| AK015427 | ― | ― |
| BC147402 | ― | ― |
| AK017005 | RIKEN cDNA 4933431I19 gene | *4933431I19Rik* |
| XM_001474011 | similar to granulocyte-macrophage colony stimulating factor receptor low-affinity subunit | *LOC100045292* |
|
| NM_010024 | dopachrome tautomerase | *Dct* |
| XM_001474007 | hypothetical protein LOC100047485 | *LOC100047485* |
| NM_001164802 | solute carrier family 38, member 10 | *Slc38a10* |
| XM_001001131 | hypothetical protein LOC677171 | *LOC677171* |
| NM_177182 | basic helix-loop-helix family, member a9 | *Bhlha9* |
| XM_001477517 | similar to E74-like factor 4 (ets domain transcription factor) | *LOC100047149* |
|
| AK006216 | ― | ― |
